# Derivational Morphology Training in French-Speaking 9- to 14- Year-Old Children and Adolescents With Developmental Dyslexia: Does It Improve Morphological Awareness, Reading, and Spelling Outcome Measures?

**DOI:** 10.1177/00222194231223526

**Published:** 2024-02-07

**Authors:** Estelle Ardanouy, Pascal Zesiger, Hélène Delage

**Affiliations:** 1 Université de Genève, Switzerland

**Keywords:** intervention, morphology, literacy

## Abstract

Children with developmental dyslexia (DD) display partially preserved morphology skills which they rely upon for reading and spelling. Therefore, we conducted explicit and intensive training of derivational morphology in French and Swiss individuals with DD, ages 9 to 14 years, in order to assess its effect on: morphological awareness, reading (speed and accuracy), and spelling. Our pre–posttest design included a group trained in derivational morphology and a group of children who continued their business-as-usual rehabilitation program with their speech-language therapist. Results showed effects on morphological awareness and on the spelling of complex words, with a large between-group effect size for trained items and a large to moderate effect size for untrained items. All these gains tended to be maintained over time on the delayed posttest, 2 months later. For reading, the results were more contrasted, with large between-group effect sizes for accuracy and speed for trained items, reducing to a small effect for accuracy on the delayed posttest. For untrained items, small effects were observed on accuracy (at both posttests) but not on speed. These results are very promising and argue in favor of using derivational morphology as a medium to improve literacy skills in French-speaking children and adolescents with DD.

Children and adolescents with developmental dyslexia (DD) have disabilities in reading accuracy and fluency, as well as in spelling and decoding (Lyon et al., 2003) which persist until adulthood (Tops et al., 2014). The DD affects between 2% and 17.5% of children depending on the study (Di Folco et al., 2021). This variability is related both to the diagnostic criteria chosen to define the disability and to the consistency of the orthography considered. Indeed, the prevalence varies between 4.5% to 12% in the United States (Lindgren et al., 1985) and 3.5% to 6.6% in France (Di Folco et al., 2021). The degree of consistency corresponds to the regularity of the relation between the phoneme and the grapheme (and vice versa) within a given orthographic system. Some orthographies are inconsistent (or opaque) in both directions, that is, in reading and spelling (e.g., English); others are mixed, like French, which is rather consistent (or transparent) in reading but opaque in spelling; finally, some other orthographic systems are fully transparent (e.g., Italian and Spanish).

There is a consensus within the scientific community regarding the fact that the core deficit underlying DD is phonological in nature, whereas possible deficits affecting other cognitive processes remain debated (Saksida et al., 2016; Wagner & Torgesen, 1987). Numerous training programs targeting phonological skills, and phonological awareness in particular, have been shown to be effective, but they do not always allow children with DD to reach a functional level of literacy (Galuschka et al., 2014). By contrast, morphological awareness skills have received less attention so far, whereas they appear to be well-developed in children with DD, which has led some authors to suggest that they may act as a compensatory mechanism (Georgiou et al., 2023). The objective of our study is to determine the effectiveness of a derivational morphology training program with French-speaking children and adolescents with DD ages 9 to 14 years on their literacy skills. Previous studies of morphology training were conducted on children who did not always have a diagnosis of DD (Bowers et al., 2010; Goodwin & Ahn, 2010) and who were primarily English-speaking. However, the morphological complexity is different between French and English (see Borleffs et al., 2017). French is considered as a morphologically rich language contrary to English which is rather morphologically sparse (Mousikou et al., 2020). The index of morphological complexity computed with the Linguistica scale is 23.05% for French against 16.88% for English (Bane, 2008). Duncan et al. (2009) further estimated that 170 suffixes existed in French, whereas only 50 were used in English. These same authors also claimed that derivation was less prevalent and less productive in English because of its Germanic roots. As for another important difference, there is a natural tendency in French to lengthen the last syllable of the word which accentuates the salience of the suffix, while this is not the case in English. Finally, suffixation brings phonological and/or orthographic changes in the word base, both in English (e.g., industry, industrial) and French (e.g., pluie [rain], pluvieux [rainy]), but this process is more dependent on lexical stress in English (Deacon et al., 2017; Duncan et al., 2009). Given these linguistic differences, we chose to conduct a study with French-speaking children and adolescents with DD.

## Derivational Morphology and Literacy

Morphology is a linguistic field that considers the smallest units of meaning in complex words, namely morphemes, and their possible combinations. More precisely, derivational morphology allows the creation of new words from a root by adding a prefix or a suffix. For example, we can add a prefix un- and a suffix -able to the root “read” to make the word “unreadable”. Morphological awareness—the conscious analysis and manipulation of morphemes—is a core skill that contributes to the child’s development in different domains such as vocabulary (Crosson & McKeown, 2016), reading fluency and comprehension (Desrochers et al., 2018; Duncan, 2018; Levesque & Deacon, 2022), as well as spelling (Deacon et al., 2009; Pacton et al., 2018).

Levesque and colleagues (2021) proposed a theoretical model, the *Morphological Pathways Framework*, which highlights the direct or indirect contribution of morphological awareness to literacy development, whether it be reading, spelling, or text comprehension. In this model, morphological awareness is located in the linguistic system, the knowledge of the orthographic representations of morphemes is in the orthographic system (Lexical Quality Hypothesis; Perfetti & Hart, 2002), and the morphological structure of words is part of the lexical representations. The authors identified three different pathways. The first is indirect and connects morphological awareness to reading decoding through morphological decoding. If the morpheme representations are of good quality, they allow for better decoding of reading, which is based on morphological units. The second indirect pathway links morphological awareness via morphological analysis to lexical representations. Morphological awareness improves lexical representations due to the semantic and syntactic information carried in morphemes. More precisely, the information which is contained in morphemes allows better prediction and recognition of words as well as better comprehension of the text. As for the last pathway, it is a direct pathway that links morphological awareness, text comprehension, and text generation.

Concerning reading, several studies have shown that children’s performance in morphological awareness tasks in Grade 2 predicts an independent share of variance in word and pseudoword reading in Grades 3, 4, and 5, after controlling for phonological awareness skills (Deacon & Kirby, 2004; Kirby et al., 2012). Further studies have shown that morphological awareness skills strongly predict decoding in Grades 4, 5, and 6, while the contribution of phonological awareness decreases from third grade (Singson et al., 2000). More precisely, a recent paper by Levesque and Deacon (2022) clarified the relationship between morphological awareness and the decoding of morphologically complex words in a longitudinal study of children followed from third to fourth grade. Results showed that morphological awareness predicted significant and unique gains in decoding complex words but not monomorphemic words. In a review paper, Duncan (2018) argued that the link between morphological awareness and decoding was causal, with evidence stemming from morphology training studies on reading (Goodwin & Ahn, 2013), as well as from longitudinal studies showing the predictive link between morphological awareness and reading (Levesque & Deacon, 2022). In another study conducted on French, Casalis, and colleagues (2009) further showed that morphological awareness adds support for visual word recognition, especially for complex words. In this way, several authors have highlighted that in dual-route reading models (Coltheart, 2006; Coltheart et al., 2001), the lexical route can be accelerated by having precise morpheme representations (Grainger & Ziegler, 2011; Marslen-Wilson et al., 1994). More precisely, the Grain Size Theory (Grainger et al., 2012) postulates that the lexical route includes either direct access to the word or indirect access through knowledge of smaller units, such as morphemes, that are directly read globally and not decoded. This model stipulates that children master larger units than phonemes to help them recognize words. Finally, morphological and reading skills appear to enrich and influence each other (Deacon et al., 2013), and morphology also contributes to reading comprehension (see Levesque et al., 2017, 2019).

With respect to spelling, several studies have found a link between morphological awareness abilities and spelling skills (Casalis et al., 2011; Deacon et al., 2009; Fejzo, 2016). For instance, the longitudinal study by Deacon and colleagues (2009) showed, in 115 children, a predictive relationship between morphological awareness skills at Grade 2 and children’s general spelling skills at Grade 4, with 8% of the variance explained, controlling for nonverbal and verbal mental skills, lexical access speed (RAN), phonological awareness, and phonological short-term memory. Other authors have shown that school-age children use their morphological skills to write morphologically complex words more accurately (Casalis et al., 2011; Pacton & Deacon, 2008; Sénéchal et al., 2006). Moreover, Pacton and colleagues (2018) demonstrated the implicit use of morphology in learning the spelling of new words in third- and fifth-grade children. In their study, children were asked to read stories with a nonword (^*^féband) in three conditions: opaque (nonwords included in stories without related words), orthographic, and morphological. In the last two conditions, the target nonword was associated with two related nonwords: two nonwords with affixes that exist in French (e.g., ^*^fébande and ^*^fébandage) for the morphological condition, and two nonwords with pseudoaffixes-letter sequences that do not form a legal affix in French (e.g., ^*^fébanduse and ^*^fébandior) for the orthographic condition. Results showed that the morphological condition was the one in which children best retained the spelling of the learned nonwords, which provides evidence for the role of morphology in spelling acquisition.

## Derivational Morphology and DD

As stated above, the phonological deficit hypothesis is the most commonly accepted explanation for the difficulties encountered in DD (Saksida et al., 2016; White et al., 2006). Although multifaceted, the phonological impairment in dyslexia is robust and persists into adulthood, even in university students who have developed functional reading skills (Cavalli et al., 2017; Martin et al., 2010). Interestingly, despite poor performance by individuals with DD on phonological tasks, their performance in morphological awareness tasks is identical to typically developing readers matched on age, vocabulary, education level, and nonverbal IQ (Cavalli et al., 2017). Furthermore, when morphological awareness tests are presented orally, young adults with DD have a higher level of morphological awareness than participants with the same reading level (Martin et al., 2014).

With respect to children, the literature suggests a developmental delay in morphological awareness skills for children with DD compared to age-matched children but similar morphological skills to those of younger children matched on reading level (Deacon et al., 2016; Georgiou et al., 2023). More specifically, the meta-analysis by Georgiou et al. (2023) showed that children with DD had a deficit in morphological awareness compared to typically developing children of the same age (*g* = 1.11). In contrast, children with DD exhibited similar scores to a group of younger, typically developing children matched on reading level (*g* = −0.08). In French, Casalis and colleagues (2004) showed that a group of children with DD, ages 8 to 12 years, obtained lower scores on all morphological awareness tasks compared to a group of age-matched children. However, when the DD group was compared to a group matched on reading level, results were task-dependent. Participants with DD scored lower on two morphemic segmentation tasks (blending or removing a base and suffix), tasks that closely resemble phonological awareness tasks. By contrast, they scored equally well on a sentence completion task (e.g., politeness/this boy is . . . polite) and on a complex word fluency test (i.e., provide words of the same family). The recruitment of phonological skills, in addition to morphological awareness, for specific tasks, may explain some of these differences. For example, to remove or add an affix, children are likely to analyze the phonological structure of the word in relation to its morphological structure. This task-dependent effect was also found in Berthiaume and Daigle’s (2014) study. They also added that French-speaking children with DD ages 9 to 12 years were sensitive to the semantic properties of morphemes.

In addition, Quémart and Casalis (2015) conducted a study in which three groups of children were asked to read a target word preceded by a prime word: a group of children with DD, a control group matched on chronological age (13 years), and a younger control group matched on reading level (9 years). When the prime was a word morphologically related to the target (e.g., farmer before farm), a significant effect of priming was found for all groups. When the prime was only semantically related (e.g., garment before dress), no priming effect was found. Finally, when the priming was orthographically analogous to the target but did not belong to the same morphological family (e.g., abricot [apricot] before abri [shelter]), no priming effect was found in the dyslexic group, whereas it was observed in both control groups. These results suggest that children with DD do not accurately code the identity of letters within words but rather coarse-grained orthographic units, that is, whole morphemes.

All of these results point to a possible compensatory mechanism of morphology in dyslexic children and adults and make it possible to exclude a causal (morphological) deficit for reading disabilities, as is the case for phonological processes (Georgiou et al., 2023). One piece of evidence that supports this compensatory morphology hypothesis is that children and adults with DD can acquire a reasonable level of reading fluency proficiency, while still experiencing a phonological disorder (Casalis et al., 2004; Cavalli et al., 2017). If morphology can be a compensatory mechanism for individuals with DD, it may be worth training this component to indirectly reinforce literacy skills.

## Training in Morphology

### Children With Typical Development

Several systematic reviews have examined the effect of morphology training on different components of literacy. The most recent meta-analysis is that of Goodwin and Ahn (2013), who synthetized the results of 6042 English-speaking children from preschool through ninth grade. The authors found moderate effects of morphology training on morphological knowledge (*d* = 0.44), phonological awareness (*d* = 0.48), vocabulary (*d* = 0.34), decoding (*d* = 0.59), and spelling (*d* = 0.30), but none on comprehension and reading fluency. Similar results were reported in the earlier review by Bowers et al. (2010), which included children (preschool to eighth grade, *N* = 2,652) who used an alphabetic orthography, with the majority being English-speaking children (18 of 22 studies). More precisely, the children trained in morphology, when compared to control groups that received regular instruction in class, progressed in their morphological processes (*d* = 0.65), but also in phonology (*d* = 0.34), reading (*d* = 0.41), spelling (*d* = 0.49), and vocabulary (*d* = 0.35). The novel aspect of this review was to differentiate the effects obtained with morphological training from those obtained with no training (i.e., control groups) or with alternative training (e.g., phonological training). Compared to alternative treatments, the morphology training had smaller, yet significant effects in morphological processes (*d* = 0.51), phonology (*d* = 0.08), reading (*d* = 0.05), spelling (*d* = 0.05), and vocabulary (*d* = 0.20). These results show that, even when compared with training programs scientifically validated by the literature (e.g., phonological training), morphology training offers similar, or even greater, gains. Furthermore, morphology interventions were found to be effective within the first hour of practice and were equally effective when training was conducted in a large group (*d* = 0.29), a small group (*d* = 0.35), or in an individual (*d* = 0.42) setting (Goodwin & Ahn, 2013). Finally, the effects were equivalent regardless of the age of the children (young or older), even if a tendency for a greater effect in younger children has been observed (Bowers et al., 2010; Goodwin & Ahn, 2013).

There are few studies on morphological training in French typically developing children. Of the studies that exist, there are two (Ardanouy et al., 2023; Casalis et al., 2018) which report moderate to large effects of classroom morphology training on morphological awareness and spelling in large groups of third and fourth graders. These effects lasted over time (i.e., were present at delayed posttest, and 3 and 5 months later) and generalized to untrained items (roots and affixes).

### Children With Reading Disabilities

The meta-analysis of Goodwin and Ahn (2010) focused exclusively on morphological training in children (kindergarten to Grade 12) with literacy disabilities (*N* = 15 to 261, depending on the study, 10 out of 17 English studies). The effects of training were moderate to low in phonological awareness (*d* = 0.49), morphological awareness (*d* = 0.40), vocabulary (*d* = 0.40), reading comprehension (*d* = 0.24), and spelling (*d* = 0.20). Furthermore, better effects were found when several strategies were used to improve literacy, that is, morphology training and another component (such as comprehensive instruction), and when training involved semantic explanation. Contrary to what had been observed in typically developing children (Goodwin & Ahn, 2013), a minimum of 10 hr of training was required for training effects to appear. Reed’s narrative review (2008) added that treating roots in morphology training, and not just affixes, improved the results. Moreover, the recent meta-analysis by Galuschka and colleagues (2020) addressed the most effective interventions for spelling remediation in dyslexics. Morphology interventions (10 of 34 studies) showed large effects on spelling (*d* = 0.80) and more moderate effects on reading (*d* = 0.30). The difference in effect size, on spelling performance, between the meta-analysis of Goodwin and Ahn (2010) and that of Galuschka and colleagues (2020) may be explained by the fact that the more recent meta-analysis also considers interventions that include work on the syllable in addition to morphological training. As for the age of the trained participants, Goodwin and Ahn (2010, 2013) and Bowers et al. (2010) reported that large effects were present for younger or older children. Galuschka et al. (2020), for their part, found effects in upper elementary and secondary grades. However, they recommended that morphological instruction begin as early as possible in the child’s curriculum, as soon as the child has mastered basic phoneme-grapheme correspondences (and vice versa). Bowers et al. (2010) added that greater effects of morphology training were found in children with the lowest levels of reading ability. Nevertheless, it is important to note that in a large majority of studies, the children do not have a diagnosis of dyslexia but are considered “poor readers” (Bowers et al., 2010; Goodwin & Ahn, 2010; Reed, 2008). Kirby and Bowers (2017) further showed the importance of explaining the phonological and orthographic changes associated with the addition of affixes (e.g., fleuve [river] becomes fluvial). The addition of an affix to a base can therefore lead to a morphological shift on the base. These derived words have been shown to be more difficult to read than morphologically transparent words in typically developing and in reading-disabled children (Kearns et al., 2016; Steacy et al., 2022). In sum, training morphological awareness seems to be a worthwhile approach for improving the skills of children with literacy disabilities.

## Aims of the Study

Research on morphological skills training in dyslexic children is still quite recent and, to the best of our knowledge, no group intervention studies have been published in French-speaking children with DD. As mentioned earlier, the consistency of orthographies and morphological properties differ from one language to the other, which underlines the importance of carrying out a study in French when the majority of published studies include English speakers. Therefore, this study reports on a derivational morphology training program in French-speaking children with DD. The predictions are as follows:

- We expect a significant improvement of trained items (*learning effect*) in morphological awareness, derivational word spelling (on roots and affixes) and reading (speed and accuracy).
- As for a *generalization effect*, we anticipate a significant improvement of the untrained items on morphological awareness, spelling of complex words (on roots and affixes) and reading (speed and accuracy). We assume that children will become aware of the morpheme units in words and will be able to segment complex words into linguistic units (base-affixes) that they know how to read or spell (Arnbak & Elbro, 2000; Casalis et al., 2018). Children are expected to learn a strategy to decompose words, which can be applied to untrained words. In this way, words will be read and spelled more accurately if the children with DD use larger linguistic units such as morphemes (Grainger et al., 2012), given their well-known difficulties with phonemes.
- We also expect a *transfer effect* to phonological skills, since the two metalinguistic components (phonological awareness and morphological awareness) share common processes. Morphological awareness and phonological awareness are indeed related skills which correlate (between .51 and .58, for example, in Carlisle & Nomanbhoy, 1993; Deacon & Kirby, 2004). More precisely, morphemes are an association of phonemes. To provide morphological instruction is to work on the form and the meaning, through a morphological and phonological analysis. A large majority of derivations result in phonological changes, which means being aware of these changes. Moreover, several meta-analyses have shown an improvement of phonological awareness skills following an intervention in morphology (Goodwin & Ahn, 2010, 2013) leading to gains on the spelling of phonologically plausible words (Arnbak & Elbro, 2000).
- Finally, maintenance of the progress should be observed over time (a *long term effect*; Casalis et al., 2018).

## Method

### Participants

A total of 86 children with DD between the ages of 9 and 14 years (*M*_age_ = 10;10) were recruited. We obtained approval for this study from the University of Geneva Ethics Committee and the parents provided written informed consent for their child’s participation. Recruitment took place by speech and language therapists (SLTs) in France and Switzerland. Indeed, a diagnosis of dyslexia had to be established by an SLT for a child to be included in the study. Several children dropped out of the study, due to change in location or parents no longer wishing their child to participate, leading to the final sample size of 82 children. Participants were randomly assigned to one of two groups, each containing 41 children. The first group was the immediate training group (ITG), corresponding to the target group that received morphological training (19 girls, four bilinguals). The second group was the delayed training group (DTG) in which children continued their business-as-usual speech and language therapy (22 girls, three bilinguals). Table 1 shows the characteristics of the two groups which were matched on age, reading age, gender, and language status. They did not differ on the following measures: vocabulary level, nonverbal reasoning, spelling, and reading (see Table 1). Among the bilingual children, the languages they spoke at home were English (2 children), Spanish (2), Turkish (2), Arabic (1), Italian (1), and Dutch (1). All children in our sample learned to read and write in French only. Finally, among the associated disorders, we found attention deficit hyperactivity disorder (11 children), developmental language disorder (9), dyscalculia (3), dyspraxia (3), and dysgraphia (2). Comorbidities are very common in DD (Peterson & Pennington, 2012; Snowling, 2012).

**Table 1.** Characteristics of the Participant Groups According to Age, Gender, Language Status, and Results on General Measures.

Variable	ITG *n* = 41	DTG *n* = 41	*T*-test or χ² value	*p*
	*M* (*SD*)	*M* (*SD*)		
Age	130.5 (17.1)	130.7 (19.1)	0.04	.97
Gender				
Female	19	22	0.44	.51
Male	22	19		
Language status				
Monolingual	37	38	0.16	.69
Bilingual	4	3		
Comorbid disorders	14	10	0.94	.33
Vocabulary, standard test (EVALEO, maximum = 100)	73.9 (13.7)	73.4 (13.3)	0.30	.76
Nonverbal reasoning, standard test (Raven matrices, maximum = 36)	30.2 (3.4)	30.6 (3.6)	0.54	.59
Spelling, standard test (Chronosdictées—*N* errors)	63.4 (17.3)	55 (25.3)	1.76	.08
Reading, standard test (EVALEO—*N* words correctly read)	133.7 (53.0)	126.5 (63.7)	0.56	.58

*Note.* ITG = immediate training group; DTG = delayed training group; EVALEO = Evaluation du Langage Ecrit et du Langage Oral. (Evaluation of Written and Oral Language).

### Materials

#### Procedure

Pre- and posttests were conducted by four trained students and by the first author. The pretest (T1) took place the week before training began. The immediate posttest (T2) was held 1 week after the end of the training, and the delayed posttest took place 10 weeks after the end of the intervention (T3).

#### Control Tasks

##### Expressive Vocabulary

We used the EVALEO test (Launay et al., 2018), a standardized computerized picture naming test, to assess children’s vocabulary range, in order to match our groups (see Table 1). We asked the children to name the pictures they saw on the screen. The maximum score was 100 (64 nouns, eight adjectives, and 28 verbs). Cronbach’s alpha given by the test was .87.

##### Nonverbal Reasoning

Raven’s progressive matrices (Raven et al., 1998) were used to measure nonverbal cognitive skills. This test allowed us to make sure that all the participants were within the typical range (above the 10th percentile) and that the two groups had a similar performance (see Table 1).

#### Morphological Awareness

Morphological skills were only tested orally in order to avoid the possibility that the children’s reading level had an impact on their performance (Duncan, 2018; Mahony et al., 2000). Given the lack of standardized tests to assess morphological awareness in French, we created three different tasks in order to test a wide range of morphological skills in our participants.

The first test was a *segmentation task*. Children had to find the root within a complex word. For example, in the word animalerie [pet shop], the child had to say animal. This task is composed of 20 items: 10 items trained in ITG and 10 untrained items. Both lists of trained and untrained words were matched on the number of syllables (*M* = 3.4) and the number of prefixes and suffixes (12 in each list). Each item, whose root was identified, received one point. Cronbach’s alpha was .67.

The second test was an *odd one out task*. The children had to decide which among three words was not derived like the others. For example, out of the three French words janvier [January], abricotier [apricot tree], and avocatier [avocado tree], janvier is the odd one out since this word is not composed of the suffix -ier/-er which means “tree.” The test is composed of 20 series of three words: one list of 10 trained in ITG and one of 10 untrained. Both lists were matched on the number of syllables (*M* = 2.74) and the number of prefixes and suffixes (five prefixes and five suffixes for each list). Each correct answer received one point. Cronbach’s alpha was .67.

The third test was a *pseudoword definition task* in which children had to find which of four pseudowords most closely matched a given definition. Pseudowords were composed of an invented root with an existing affix. For example, “to be extremely *goulé” was *archigoulé (as opposed to *sous-goulé, *prégoulé, and *cogoulé) because the prefix “archi-” means “extremely” in French. The test was composed of 25 definitions: 10 concerning prefixes and 15 concerning suffixes. None of these items were included in the intervention. The possible answers for each definition were also proposed in writing, in addition to the oral modality, to avoid an overload of verbal working memory. Each correct answer received one point. Cronbach’s alpha was .73.

#### Reading

##### Standardized Reading Test

The children had to read the text “The Seagull” from the EVALEO standardized test (Launay et al., 2018) to measure reading fluency. The score was the number of words read correctly in 2 min. This test allowed us to match our groups on reading level (see Table 1).

##### Experimental Reading Task

For this study, we constructed several matched word lists. The A list corresponded to complex words trained in ITG (10 words with a prefix and 10 words with a suffix), to test for a learning effect. The B list corresponded to untrained complex words administered to both groups (10 words with a prefix and 10 words with a suffix), to test for a generalization effect. The affixes were the same between List A and List B. For example, the prefix “para-” was studied in the word parapente [paraglider] in the A list, but the B list used the word parachute. This B-word list existed in two versions, B1 and B2, to avoid a test–retest effect. Specifically, the A list had nine words with a morphological shift (and 11 morphologically transparent) from the base at the phonological level (i.e., causing a difference in pronunciation: Egypte [Egypt], Egyptien [Egyptian]). In each of the B1 and B2 lists, there were 11 words with a morphological shift. Each list of words (A, B1, and B2) yielded a reading speed score and an accuracy score. These word lists were matched on the number of syllables (*M* = 3.05; *p* = 1), graphemes (*M* = 7.92; *p* > .81), letters (*M* = 8.93; *p* > .82), reading consistency (*M* = 77.41; *p* > .52), and frequency (*M* = 4.23; *p* > .28) according to the *Manulex-infra* database (Peereman et al., 2007). Details of the lists are provided in Supplemental Appendix A. Cronbach’s alpha for Lists A, B1, and B2 were .77, .75, and .72, respectively.

#### Spelling

##### Standardized Spelling Test

We used a dictation of sentences from the standardized test “Chronosdictées” (Baneath et al., 2015). The children had to write between five and eight sentences according to their grade (third grade: five sentences for a total of 60 words; fourth grade: seven sentences/87 words; fifth grade: six sentences/82 words; sixth grade and above: eight sentences/124 words). This test allowed us to calculate several different scores: number of lexical spelling errors, number of inflectional errors, number of phonologically plausible errors, number of segmentation errors (e.g., the bad segmentation of the word *la viateur/l’aviateur [aviator]), number of word omissions, and a total error score. This last score allowed us to ensure that the two groups of children were matched on their general spelling level (see Table 1). We performed a double scoring of all the dictations and the inter-judge agreement was 96.2%.

##### Experimental Spelling Task

Based on the same model as the reading task, we created different lists (A, B1, and B2). These word lists were matched on the number of syllables (*M* = 3.1; *p* > .17), graphemes (*M* = 7.6; *p* > .45), letters (*M* = 9.15; *p* > .17), spelling consistency (*M* = 74.8; *p* > .59), and frequency (*M* = 4.35; *p* > .85) according to the *Manulex-infra* database (Peereman et al., 2007). Specifically, List A had nine words with a morphological shift (and 11 morphologically transparent words) from the base at the orthographic level (i.e., resulting in a difference in the spelling of the base: histoire [history], historique [historical]). For Lists B1 and B2, there were 11 words with a morphological shift. We attributed one point per well-written affix and one point per well-written root, each word thus yielding a maximum of two points. We double-scored all word dictations, and the inter-judge agreement score was 99.1%. Details of the lists are provided in Supplemental Appendix B. A, B1, and B2 lists scored .84, .85, and .86 for Cronbach’s alphas, respectively.

### Intervention

#### Procedure

Training was conducted either by SLTs or students who were themselves trained in the morphological intervention protocol or by the first author. All the experimenters received a 2-hr training on how to conduct the intervention with the children. The experimenters followed a checklist for every session regarding the steps to be followed and the time to be allocated to each step: (a) introduction of the example words; (b) explicit teaching time related to the day’s session (e.g., meaning of the affixes trained on and how to extract the root of the word); (c) details of the application exercises and correction with the points on which to draw the children’s attention (e.g., the spelling change of the base between the simple word and the derived word). To ensure fidelity of the intervention, the experimenters were asked to check off the steps performed, to note the session date and the time actually allocated for each step but also to provide the exercise booklet completed by each child. Moreover, for each exercise, the instructions and the example were explained by the adult; then, the child read the items, with feedback from the adult if the reading was incorrect; finally, the child systematically wrote the answer, also with feedback if necessary.

The training was conducted individually and took place over a period of 10 to 12 weeks, twice a week, for a total of 20 sessions to be adapted to the clinical reality of the SLTs. It lasted between 10 and 15 hr, depending on the child’s speed (30–45 min per session). The person who performed the pre- and posttests was different from the person who conducted the training, without being blind to whether the children had completed the training or not.

#### Immediate Training Group

The morphological training was the same as the one described by Ardanouy et al. (2023). It was based on the intervention program employed in Carlisle’s (2010) study and was provided in oral and written modalities (as advocated by Nunes et al., 2003). The list of affixes trained was selected within the *Polymots* database (Gala & Rey, 2008) on the basis of the frequency of occurrence in French. Then, the words containing these specific affixes were chosen in the *Manulex-infra* database (Peereman et al., 2007) as a function of their frequency of occurrence in French primary school textbooks. The word lists for the pre- and posttests were matched. The derived words trained were, therefore, those with the most frequent affixes in words frequently encountered by elementary school children to fit in with what will be most useful for children’s literacy.

In the first four sessions, we explained to the child what derivational morphology was and we worked specifically on word roots (as recommended by Reed, 2008). The children then learned how to divide complex words into prefixes, roots, and suffixes. The exercises focused on extracting the roots of words, and comparing words in which the root had not been changed (i.e., redire [retell]/ dire [tell]) or had been changed (i.e., verdâtre [greenish]/ vert [green]). We also explained to the children that the root could change orthographically and/or phonologically when prefixes or suffixes were added to help them read words with morphological shifts that are particularly complex to master (Kearns et al., 2016; Kirby & Bowers, 2017; Steacy et al., 2022). The next five sessions focused on learning 18 different prefixes and Session 10 was a review session. The next seven sessions were dedicated to 26 different suffixes. Finally, the last three sessions were again review sessions.

All the sessions started with an introduction in which children had to guess from a few words which affixes were going to be focused on in that lesson (two to five affixes per session), and then they had to try to deduce the meaning of the affix. A series of exercises was then proposed: segmentation exercises, in which the root was written down; sentence completion exercises with affixed words or pseudowords (i.e., write the answer in the following sentences: “The person who cycles is a . . . cyclist”; “the person who does ^*^fourbi is a . . . ^*^fourbist”); word family exercises (i.e., write as many words in the family as possible); odd one out exercises (i.e., circle the word that is not complex) and finally multiple-choice questions with words or pseudowords (i.e., from a given definition, choose the corresponding word). For example, for the item: a ^*^mitainerie [^*^mittenshop] is (1) a small mitten, (2) a person who makes mittens, (3) the opposite of a mitten, (4) the place where mittens are sold; Answer 4 was expected. Specifically, the items used in the morphological awareness tests were presented between five and eight times each for the trained list. The words in the reading and spelling trained lists (A lists) were presented between eight and 12 times each during the intervention. Affixes were presented in at least two different words (e.g., découpage [cut] and recyclage [recycle]) to show the child that they can be used in different words.

#### Delayed Training Group

The children of the group that did not follow the training continued their speech and language therapy sessions at the same rate as the training group. We asked the speech therapists not to work specifically on derivational morphology, although the sessions were likely to include activities around reading and spelling. At the end of the posttest phase, we provided the training material to the SLTs so that the children in the DTG could complete the training as well.

## Results

### Data Analysis

In both groups, the distribution of scores in morphological awareness, reading, and spelling was close to normal for T1, T2, and T3. Progress in morphological awareness, reading, and spelling was analyzed between pretest and posttests with a 2 × 3 repeated-measures analysis of variance (ANOVA) design, with Group as between-subject factor (ITG vs. DTG) and Time as within-subject factor (T1, T2, and T3). To further examine the nature of the effects, we conducted within-group Bonferroni post hoc tests. Finally, we quantified inter-group effect sizes using Cohen’s *d* (where *d* = $\left(M_{2}-M_{1}\right)/\sqrt{\left(SD_{1}^{2}-SD_{2}^{2}\right)/2)}$ ).

### Morphological Awareness

For the morphological awareness tests, we summed the items trained together (10 items from the segmentation task and 10 items from the odd one out task), which gave us a score out of 20 points for the items trained in morphological awareness for ITG. In the same way, the untrained items were added together to give a score out of 20 points. The means and standard deviations of the scores on all morphological awareness tests are presented in Table 2, and the results of the ANOVAs for the morphological awareness tests are summarized in Table 3. For all morphological awareness tests, ANOVA results revealed main effects of Time, of Group, and a Time × Group interaction. We conducted post hoc group comparisons for each time point, which are detailed below.

**Table 2.** Means and Standard Deviations of Results on Morphological Awareness Tests in the ITG and DTG Groups at Different Times (T1, T2, and T3).

Measure	Time	ITG		DTG	
		*M*	*SD*	*M*	*SD*
Trained items	T1 (*n* = 82)	10.37	2.34	10.95	2.89
Maximum = 20	T2 (*n* = 82)	17.20	2.37	10.90	2.40
	T3 (*n* = 77)	16.43	2.98	11.45	3.19
Untrained items	T1 (*n* = 82)	8.22	2.48	8.90	2.75
Maximum = 20	T2 (*n* = 82)	12.02	3.53	8.76	2.90
	T3 (*n* = 77)	12.30	3.50	9.15	2.77
Pseudoword definition task	T1 (*n* = 82)	12.02	4.52	12.76	4.19
	T2 (*n* = 82)	18.98	3.66	13.41	4.68
Maximum = 25	T3 (*n* = 77)	16.76	6.65	14.92	5.30

*Note.* ITG = immediate training group; DTG = delayed training group.

**Table 3.** Results of Repeated-Measures ANOVAs on Morphological Awareness Tasks.

Measure	Main effects	*F*	η^2^
Trained items	Time	*F*(2, 150) = 70.3***	.48
	Group	*F*(1, 75) = 46.3***	.38
	Time × Group	*F*(2, 150) = 69.3***	.48
Untrained items	Time	*F*(2, 150) = 13.0***	.14
	Group	*F*(1, 75) = 8.0**	.09
	Time × Group	*F*(2, 150) = 15.4***	.16
Pseudoword definition task	Time	*F*(2, 150) = 29.1***	.27
	Group	*F*(1, 75) = 6.3*	.07
	Time × Group	*F*(2, 150) = 16.5***	.17

* *p* < .05. **p < .01. ***p < .001.

#### Trained Items

In the ITG, a significant difference was noted between T1-T2, *p* < .001 and T1-T3, *p* < .001, but no difference was found between T2 and T3, *p* = 1. In the DTG, no significant difference was observed, *p* = 1 for all comparisons. Between-group effect sizes for both posttests were large, *d* = 2.64, for the immediate posttest and *d* = 1.61 for the delayed posttest.

#### Untrained Items

For the ITG, we also found a significant difference between T1 and T2, *p* < .001, and between T1 and T3, *p* < .001, and no difference for T2-T3, *p* = 1. In the DTG, we observed no difference, *p* = 1 for all comparisons. Between-group effect sizes for both posttests were large, *d* = 1.01, for the immediate posttest, and *d* = 1.00 for the delayed posttest.

#### Generalization: Pseudoword Definition

Here again, post hoc group comparison revealed a significant difference between T1 and T2, *p* < .001, and T1-T3, *p* < .001 while a nonsignificant difference was observed for T2-T3, *p* = .07. No significant difference was noticed in the DTG, *p* > .09 for all comparisons. Between-group effect sizes were large for the immediate posttest, *d* = 1.33, and small for the delayed posttest, *d* = 0.31.

In summary, the results showed an improvement, only in the trained group (ITG), on all morphological awareness tests. The ITG children performed significantly better on both posttests (immediate posttest and delayed posttest) compared to the pretest for the trained items, the untrained items, and the pseudoword definition task.

### Reading

Table 4 presents the means and standard deviation for the scores on reading tests. Table 5 shows ANOVA results for reading tests.

**Table 4.** Means and Standard Deviations of Results on Reading Tests in the ITG and DTG Groups at Different Times (T1, T2, and T3).

	Time	ITG		DTG	
Measure		*M*	*SD*	*M*	*SD*
List A (Trained items)	T1 (*n* = 82)	14.88	3.47	15.29	3.63
Accuracy	T2 (*n* = 82)	18.80	1.23	15.83	3.74
Max = 20	T3 (*n* = 76)	17.34	2.04	16.63	3.22
List A	T1 (*n* = 82)	55.84	21.98	65.65	28.95
Reading time	T2 (*n* = 82)	29.49	11.62	54.28	25.47
	T3 (*n* = 76)	29.92	11.98	48.87	26.08
List B (Untrained items)	T1 (*n* = 82)	15.07	3.27	15.29	3.63
Accuracy	T2 (*n* = 82)	16.80	2.64	15.82	3.74
Max = 20	T3 (*n* = 76)	17.33	2.04	16.68	3.05
List B	T1 (*n* = 82)	52.91	21.19	62.38	24.39
Reading time	T2 (*n* = 82)	44.54	20.50	58.85	27.56
	T3 (*n* = 76)	41.39	17.60	52.64	24.87

*Note.* ITG = immediate training group; DTG = delayed training group.

**Table 5.** Results of Repeated-Measures ANOVAs on Reading Tests.

Measure	Main effects	*F*	η^2^
List A (Trained items)	Time	*F*(2, 148) = 49.35***	.40
Accuracy	Group	*F*(1, 74) = 6.54*	.08
	Time × Group	*F*(2, 148) = 20.20***	.21
List A	Time	*F*(2, 148) = 88.03***	.54
Reading time	Group	*F*(1, 74) = 16.21***	.17
	Time × Group	*F*(2, 148) = 7.29***	.09
List B (Untrained items)	Time	*F*(2, 148) = 14.30***	.16
Accuracy	Group	*F*(1, 74) = 0.05	< .001
	Time × Group	*F*(2, 148) = 5.11**	.06
List B	Time	*F*(2, 148) = 18.11***	.20
Reading time	Group	*F*(1, 74) = 6.66*	.08
	Time × Group	*F*(2, 148) = 0.88	.01

* *p* < .05. **p < .01. ***p < .001.

#### Trained A List

For the accuracy and reading speed of the A list, ANOVA results showed main effects of Time, of Group, and a Time × Group interaction. Then, we performed post hoc group comparisons for each time point.

##### Accuracy

In the ITG, a significant difference was seen between T1 and T2, *p* < .001, and between T1 and T3, *p* < .001, and no difference for T2-T3, *p* = 1. In the DTG, a significant difference was observed between T1 and T3. Between-group effect sizes were large for the immediate posttest, *d* = 1.07, and small for the delayed posttest, *d* = 0.26.

##### Reading Time

In both groups, we found a significant difference between T1 and T2, *p* < .001, and T3, *p* < .001. Post hoc comparisons revealed no differences between T2 and T3 in both groups, *p* > .16. Between-group effect sizes for both posttests were large in favor of ITG, *d* = 1.25, for the immediate posttest, and *d* = 0.93 for the delayed posttest.

#### Untrained B List

##### Accuracy

The ANOVA results indicated a main effect of Time but not of Group, and an interaction effect of Time × Group. Post hoc comparisons showed a significant difference in the ITG, between T1 and T2, *p* < .001, and T1-T3, *p* < .001, and no difference between T2 and T3, *p* = 1. In the DTG, no difference was found in all comparisons, *p* = 1. Between-group effect sizes for both posttests were small, *d* = 0.30, for the immediate posttest and *d* = 0.25 for the delayed posttest.

##### Reading Time

Results of the ANOVA showed an effect of Time and of Group but no Time × Group interaction.

To sum up, the results showed a significant improvement in the performance of children in the trained group only in reading accuracy for both trained (List A) and untrained (List B) words. These results were maintained between the immediate posttest and the delayed posttest.

### Spelling

Table 6 provided the average scores for the spelling tests, and Table 7 reported the results of the ANOVAs. Average scores for whole-word scoring are provided in Supplemental Appendix C.

**Table 6.** Means and Standard Deviations of Spelling Tests in the ITG and DTG Groups at Different Times (T1, T2, and T3).

Measure	Time	ITG		DTG	
		*M*	*SD*	*M*	*SD*
List A (Trained items)	T1 (*n* = 82)	9.37	3.04	9.61	4.06
Affixes	T2 (*n* = 82)	14.76	4.02	10.02	3.64
Maximum = 20	T3 (*n* = 76)	14.06	3.24	10.55	3.64
List A	T1 (*n* = 82)	7.29	3.55	8.88	4.29
Roots	T2 (*n* = 82)	12.78	3.17	8.85	3.92
Maximum = 20	T3 (*n* = 76)	12.28	3.03	9.58	4.60
List B (Untrained items)	T1 (*n* = 82)	9.76	3.13	11.20	4.37
Affixes	T2 (*n* = 82)	14.39	3.10	10.88	3.85
Maximum = 20	T3 (*n* = 76)	14.00	2.87	11.45	4.38
List B	T1 (*n* = 82)	7.76	3.47	8.98	4.11
Roots	T2 (*n* = 82)	11.56	3.29	9.17	3.55
Maximum = 20	T3 (*n* = 76)	11.19	3.03	9.30	3.73
Nonphonologically	T1 (*n* = 82)	14.22	8.49	11.27	8.00
Plausible errors	T2 (*n* = 82)	10.80	7.55	11.71	8.42
(Chronosdictées)	T3 (*n* = 76)	10.81	7.94	10.17	8.08

*Note.* ITG = immediate training group; DTG = delayed training group.

**Table 7.** Results of Repeated-Measures ANOVAs on Spelling.

Measure	Main effects	*F*	η^2^
List A (Trained items)	Time	*F*(2, 148) = 56.03***	.43
Affixes	Group	*F*(1, 74) = 14.71***	.16
	Time × Group	*F*(2, 148) = 33.25***	.31
List A	Time	*F*(2, 148) = 50.21***	.40
Roots	Group	*F*(1, 74) = 4.95*	.06
	Time × Group	*F*(2, 148) = 40.11***	.35
List B (Untrained items)	Time	*F*(2, 148) = 45.26***	.38
Affixes	Group	*F*(1, 74) = 4.22*	.05
	Time × Group	*F*(2, 148) = 44.70***	.38
List B	Time	*F*(2, 148) = 26.08***	.26
Roots	Group	*F*(1, 74) = 2.26	.03
	Time × Group	*F*(2, 148) = 18.37***	.20
Nonphonologically	Time	*F*(2, 148) = 7.10**	.08
Plausible errors	Group	*F*(1, 74) = 0.23	<.01
(Chronodictées)	Time × Group	*F*(2, 148) = 5.78**	.07

* *p* < .05. **p < .01. ***p < .001.

#### Trained A List

For the spelling of affixes and roots of the A list, ANOVA results showed the main effects of Time, of Group, and a Time × Group interaction. As before, we conducted post hoc comparisons for each time point.

##### Affixes

In the ITG, a significant difference was reported between T1 and T2, *p* < .001, and T1-T3, *p* < .001, but no difference was found between T2 and T3, *p* = .59. In the DTG, no significant difference was observed, *p* > .28 for all comparisons. Between-group effect sizes for both posttests were large, *d* = 1.24, for the immediate posttest, and *d* = 1.02 for the delayed posttest.

##### Roots

In the ITG, we found a significant difference between T1 and T2, *p* < .001, and T1-T3, *p* < .001, whereas no difference was observed between T2 and T3, *p* = 1. In the DTG, no significant difference was seen, *p* = 1 for all comparisons. Between-group effect sizes were large for the immediate posttest, *d* = 1.10, and moderate for the delayed posttest, *d* = 0.69.

#### Untrained B List

##### Affixes

Results of the ANOVA showed an effect of Time, of Group, and a Time × Group interaction. We found significant differences between T1 and T2, *p* < .001, and T1-T3, *p* < .001, only in the ITG. Between-group effect sizes were large for the immediate posttest, *d* = 1.00, and moderate for the delayed posttest, *d* = 0.69.

##### Roots

The analyses indicated an effect of Time, but no effect of Group, and a Time × Group interaction. In the ITG, post hoc comparison revealed a significant difference between T1 and T2, *p* < .001, and T1-T3, *p* < .001. No difference was found in the DTG, *p* = 1 for all comparisons. Between-group effect sizes for both posttests were moderate, *d* = 0.70, for the immediate posttest, and *d* = 0.56 for the delayed posttest.

#### Dictation—Nonphonologically Plausible Errors

The ANOVA results showed an effect of Time, but no effect of Group, and a Time × Group interaction. In the ITG, we found a significant difference between T1 and T2, *p* < .01, and T1-T3, *p* < .01. In the DTG, no difference was observed, *p* > .53 for all comparisons. Between-group effect sizes for both posttests were small, *d* = 0.11, for the immediate posttest, and *d* = 0.08 for the delayed posttest.

To summarize the spelling results, we found a significant improvement in the spelling of trained words (affixes and roots) and untrained words (affixes and roots) between the pretest and the immediate posttest, only in the group who received the derivational morphology intervention. The results also showed a significant decrease in the number of nonphonologically plausible errors in sentences dictation, only in the ITG children. All these results were maintained at the delayed posttest.

## Discussion

The aim of the study was to evaluate the effectiveness of explicit and specific training in derivational morphology on morphological awareness, spelling, and reading in children and adolescents with DD, ages 9 to 14 years. Our main results showed a significant improvement in morphological awareness, on both trained and untrained items, in both the immediate (large between-group effect sizes) and delayed (large to small between-group effect sizes) posttests but also in the spelling of complex morphologically derived words, on both trained and untrained items, in both the immediate (large to moderate between-group effect sizes) and delayed (large to moderate between-group effect sizes) posttests. For reading accuracy, our findings revealed, for trained items, a large between-group effect size at immediate posttest, but which was reduced to a small one at the delayed posttest. For reading speed, the between-group effect sizes, for trained items, remained large across the immediate and delayed posttests. Moreover, only reading accuracy improved for untrained words, with small between-group effect sizes at both posttests. In addition, the results showed a decrease in the number of nonphonologically plausible errors in a sentence dictation task. These different findings will be detailed below.

### Learning Effect

Our first objective was to determine whether morphology training had an effect on trained items: in morphological awareness, in reading (time and accuracy), and in spelling. In *morphological awareness*, the between-group effect sizes showed a large effect on the two tests that contained trained items (segmentation and odd one out tests), which reveals that learning took place in the ITG. These results are consistent with those found in two meta-analyses (Bowers et al., 2010; Goodwin & Ahn, 2010), which showed a direct effect of morphological training on morphological skills. In the case of the *reading* of morphologically complex trained words, the results showed improvement for speed and accuracy, for trained words, with a large between-group effect size, in favor of the ITG group. Finally, for *spelling* of morphologically complex words trained in the ITG, findings showed large between-group effect sizes, for affixes and roots. Specifically, affixes were spelled better than roots, which can be explained by the fact that their meaning and spelling were learned explicitly and that children encountered them at a higher frequency in many different words than they encountered roots. These results partly support those of the randomized controlled study by Colenbrander et al. (2022), which showed improvement in the spelling skills of children with literacy disabilities after morphological training, whether explicit or implicit.

### Generalization Effect

The second objective was to investigate whether training in derivational morphology led to generalization of the learned strategy to untrained items. For *morphological awareness*, our results showed significant improvement, with large between-group effect sizes, on the untrained items of the various tasks we used (segmentation, odd one out, and pseudoword definition tests), suggesting that children with DD are able to use morphological learning to improve their performance on morphological awareness. These results corroborate partially those of Arnbak and Elbro (2000); this study is one of the few that has proposed morphology training for children with a dyslexia diagnosis. These authors predicted that morphological training would lead to widespread changes in reading and spelling, but they found improvement only on certain tests, possibly because the items they selected contained phonological modifications within the root of the words.

For *reading*, the results obtained on untrained words were different according to whether accuracy and reading speed were measured. In the case of accuracy, we found a small between-group effect size. The number of average words read increases by only 1.7 words, which is still quite modest. Moreover, no interaction effect was found for reading speed in both groups of children. The improvement in reading speed for trained items (in contrast to untrained words) thus seems to be linked more to repeated exposure to words (Share, 1999) than to the application of a morphological strategy. Therefore, morphology training seems to help children with DD read morphologically complex words more accurately but not more fluently to a slight extent. The lack of improvement in reading speed is consistent with the fact that morphological awareness allows for the development of a strategy for decoding words by meaning. The Grain Size reading theory proposed by Ziegler and Goswami (2005) emphasizes that children can use larger units to read rather than graphemes, such as the syllable or morpheme, especially in inconsistent orthographies such as English or French. However, the use of these larger units is more costly because the number of correspondences to learn is higher. This suggests that children do not use morphology systematically in reading or that they need more time to implement this reading strategy.

For the *spelling* of (untrained) morphologically complex words, results showed a significant improvement, in affixes and roots with large and moderate between-group effect sizes, respectively. This result is very interesting as it highlights the use of a morphological strategy by participants, even on untrained words. That is, children were able to segment words in order to recognize trained affixes and roots that they already knew, not from this study, but from other learning opportunities. Our results thus corroborate those of Colenbrander and colleagues (2022) who revealed progress on untrained items linked to explicit training of derivational morphology in children with literacy disabilities.

Finally, the dissociation between reading and spelling scores for the transfer effect (List B: untrained items) may be related to two factors. On one hand, the French orthographic system is already transparent for reading, so the contribution of morphology is likely to be minimal (as found in Ardanouy et al., 2023; Casalis & Louis-Alexandre, 2000) compared to spelling (Galuschka et al., 2020). On the other hand, it was difficult to control for fidelity of training for reading compared to spelling. It is therefore possible that the children did not systematically read the derived words in the training exercises, contrary to the spelling part where it is easy to check the fidelity with the written trace in the booklet.

### Transfer Effect on Phonology

The third objective was to check whether there was a transfer effect of the morphology training onto phonological skills. Several studies have shown links between morphological and phonological awareness, both domains sharing common variance but also reflecting different abilities (Carlisle & Nomanbhoy, 1993; Casalis & Louis-Alexandre, 2000; Deacon & Kirby, 2004). In our study, children in the ITG were the only ones to show a significant decrease in the number of nonphonologically plausible errors in sentence dictation. Even if the between-group effect size was small, this result highlights the links between sublinguistic units: phonological and morphological. The reasoning is as follows: by training morphological units, smaller than words, children are encouraged to focus more precisely on word composition and thus indirectly to pay more attention to other sublexical units, such as phonemes. This effect on phonological awareness was already found in several studies (Casalis & Colé, 2009; Goodwin & Ahn, 2010) or on the spelling of phonologically plausible words (Arnbak & Elbro, 2000). Specifically, Casalis and Colé (2009) reported that training in derivational morphology resulted in better phonological sensitivity but not better phoneme manipulation in kindergarten children. Our results add an interesting dimension to the value of training morphological awareness for improving spelling abilities. They also reinforce the possibility that the metalinguistic skills of morphological and phonological awareness share common processes that allow for mutually beneficial influence.

### Long-Term Effect

Finally, our last objective was to verify if the gains observed in the immediate posttest were maintained in the long term, namely in the delayed posttest, 2 months later. In the trained group, all gains, whether on trained or untrained items, were maintained over time despite a small decrease in some results (based on post hoc analysis). None of the decreases in scores between the immediate and the delayed posttest were significant. However, several between-group effect sizes change from large to moderate: for spelling of trained word roots (*d* = 1.10–0.69) and for affixes of untrained words (*d* = 1.00–0.69), where the memory effect comes into play. This effect is also found for morphological awareness, with an effect size that goes from large to small, only for the pseudoword definition task (*d* = 1.33–0.31), where we can hypothesize that the children in the control group are getting used to the task, which is not very easy at first sight. Finally, for reading accuracy, the between-group effect size went from large to small for trained words (*d* = 1.07–0.26), most likely indicating a familiarization effect of the control group to the reading task.

These results should be highlighted since children with DD are known to have poorer memorization skills than typically developing children for rote learning (Demoulin & Kolinsky, 2016; Menghini et al., 2010; Szmalec et al., 2011). For example, Binamé and colleagues (2015) found that children with DD retained the spelling of words less well than children of the same chronological age or younger children of the same reading level, despite having received an extended training (10 repetitions for each word). Thus, children with DD have difficulties in having accurate spelling representations. Teaching them a strategy such as morphological awareness that emphasizes meaning appears to be effective for these children.

### Limitations

Although our results are very promising with respect to the effectiveness of explicit training in derivational morphology on morphological awareness, reading, and spelling, our study has several limitations. First, our study included children and adolescents with DD who ranged in age from 9 to 14 years. It would have been interesting to have formed two subgroups in terms of age in order to have compared the effectiveness of training in younger and older children; however, this would have required a larger sample size. Second, it would have been interesting to have added a task of lexical comprehension to our test battery to determine if children’s vocabularies increased as a result of training (as found in Goodwin & Ahn, 2010). Third, children often lost motivation at the end of treatment. A more playful adaptation of the morphological tasks might help children maintain their interest through to the end of training. Finally, due to the lack of French standardized tests to assess morphological awareness, we had to create our own tests for this study, but their internal consistency was quite low, with Cronbach’s alphas between .67 and .70, whereas the threshold of 0.70 is recognized as being the low limit to have optimal reliability (Nunnally, 1978). Thus, these tests could be improved for future investigations and ultimately standardized for clinical use.

### Theoretical and Applied Implications

This study is the first, to the best of our knowledge, to offer derivational morphology training to French-speaking children with DD. The results suggest that morphological instruction is an effective way to improve literacy outcomes in these children. These findings highlight the potential supporting role of morphological awareness on literacy skills. They also argue in favor of the hypothesis of a causal role of morphological awareness on literacy skills and, specifically, for the current study, on spelling in children with DD. These results thus add further support to this hypothesis that is still debated as intervention studies carried out so far reported contrasting results (Casalis & Louis-Alexandre, 2000; Kuo & Anderson, 2006; Nagy et al., 2006). In addition, this study should provide impetus for teachers and SLTs to develop rehabilitative programs based on morphological awareness to improve children’s literacy skills. Further research with larger samples of children is needed to validate the effectiveness of our derivational morphology training program.

### Conclusion

Very little research has been conducted on the training of morphological skills in school-age children with dyslexia. Our study presented findings on the use of explicit training on derivational morphology, in particular, on the teaching of affix meaning, to children with DD. More precisely, this study showed the positive impact of such training on morphological awareness, spelling (large effect), and reading accuracy (lower effect) on untrained items. Thus, derivational morphology training is a tool that can be used by professionals who work with children with DD. Finally, morphological training provides a complement to phonological training that quickly reaches its limit in individuals with DD who, for the most part, retain a deficit in phonological awareness.

## Supplemental Material

sj-docx-1-ldx-10.1177_00222194231223526 – Supplemental material for Derivational Morphology Training in French-Speaking, 9- to 14- Year-Old Children and Adolescents With Developmental Dyslexia: Does it Improve Morphological Awaraness, Reading and Spelling Outcome Measures? Supplemental material, sj-docx-1-ldx-10.1177_00222194231223526 for Derivational Morphology Training in French-Speaking, 9- to 14- Year-Old Children and Adolescents With Developmental Dyslexia: Does it Improve Morphological Awaraness, Reading and Spelling Outcome Measures? by Estelle Ardanouy, Pascal Zesiger and Hélène Delage in Journal of Learning Disabilities

sj-docx-2-ldx-10.1177_00222194231223526 – Supplemental material for Derivational Morphology Training in French-Speaking, 9- to 14- Year-Old Children and Adolescents With Developmental Dyslexia: Does it Improve Morphological Awaraness, Reading and Spelling Outcome Measures? Supplemental material, sj-docx-2-ldx-10.1177_00222194231223526 for Derivational Morphology Training in French-Speaking, 9- to 14- Year-Old Children and Adolescents With Developmental Dyslexia: Does it Improve Morphological Awaraness, Reading and Spelling Outcome Measures? by Estelle Ardanouy, Pascal Zesiger and Hélène Delage in Journal of Learning Disabilities

sj-docx-3-ldx-10.1177_00222194231223526 – Supplemental material for Derivational Morphology Training in French-Speaking, 9- to 14- Year-Old Children and Adolescents With Developmental Dyslexia: Does it Improve Morphological Awaraness, Reading and Spelling Outcome Measures? Supplemental material, sj-docx-3-ldx-10.1177_00222194231223526 for Derivational Morphology Training in French-Speaking, 9- to 14- Year-Old Children and Adolescents With Developmental Dyslexia: Does it Improve Morphological Awaraness, Reading and Spelling Outcome Measures? by Estelle Ardanouy, Pascal Zesiger and Hélène Delage in Journal of Learning Disabilities
