# Supplementary material for: Derivational Morphology Training in French-Speaking 9- to 14- Year-Old Children and Adolescents With Developmental Dyslexia: Does It Improve Morphological Awareness, Reading, and Spelling Outcome Measures?
Source: J Learn Disabil. 2024 Feb 7;58(1):62–77. doi: 10.1177/00222194231223526 (PMC11636023; doi:10.1177/00222194231223526)
Supplement: sj-docx-1-ldx-10.1177_00222194231223526 – Supplemental material for Derivational Morphology Training in French-Speaking, 9- to 14- Year-Old Children and Adolescents With Developmental Dyslexia: Does it Improve Morphological Awaraness, Reading and Spelling Outcome Measures? [file sj-docx-1-ldx-10.1177_00222194231223526.docx]

**JOURNAL OF LEARNING DISABILITIES SUPPLEMENTAL FILE**

Derivational Morphology Training in French-Speaking, 9- to 14-Year-Old Children and Adolescents with Developmental Dyslexia: Does it Improve Morphological Awareness, Reading and Spelling Outcome Measures?

**Appendix A**

*Experimental reading task*

**List A**

Bilingue (bilingual), tricycle, contresens (misinterpretation), international, antirouille (anti-rust), Egyptien (Egyptian), humanité (humanity), vilain (ugly), alpinisme (mountaineering), furieusement (furiously), prétexte (pretext), monotone (monotonous), proposer (to offer), cohabitation, entrejambe (pace), éclairage (lighting), septième (seventh), épicerie (grocery), sportif (sportsperson), fluvial (river)

**List B1**

Bicoque (catamaran), trimestre (quarter), contrepoids (counterweight), intermédiaire (intermediate), antibruit (noise-reducing), Alsacien (Alsatian), humidité (humidity), forain (fairground), athlétisme (track and field), curieusement (curiously), prévision (projection), monocycle (unicycle), promettre (to promise), coexistence, entresol (mezzanine), nettoyage (cleaning), troisième (third), bergerie (sheepfold), tardif (late), matinal (morning)

**List B2**

Bicolore (two-colored), triangle, contrebasse (double bass), interlocuteur (interlocutor), antirides (anti-wrinkle), Tahitien (Tahitian), rapidité (rapidity), mondain (society), cyclisme (cycling), copieusement (copiously), préhistoire (prehistory), monocorde (monotone), provenir (to come from), coéquipier (teammate), entrefilet (short item), essayage (testing), deuxième (second), bijouterie (jeweler’s), massif (massive), central.
