# Supplementary material for: Derivational Morphology Training in French-Speaking 9- to 14- Year-Old Children and Adolescents With Developmental Dyslexia: Does It Improve Morphological Awareness, Reading, and Spelling Outcome Measures?
Source: J Learn Disabil. 2024 Feb 7;58(1):62–77. doi: 10.1177/00222194231223526 (PMC11636023; doi:10.1177/00222194231223526)
Supplement: sj-docx-2-ldx-10.1177_00222194231223526 – Supplemental material for Derivational Morphology Training in French-Speaking, 9- to 14- Year-Old Children and Adolescents With Developmental Dyslexia: Does it Improve Morphological Awaraness, Reading and Spelling Outcome Measures? [file sj-docx-2-ldx-10.1177_00222194231223526.docx]

**JOURNAL OF LEARNING DISABILITIES SUPPLEMENTAL FILE**

Derivational Morphology Training in French-Speaking, 9- to 14-Year-Old Children and Adolescents with Developmental Dyslexia: Does it Improve Morphological Awareness, Reading and Spelling Outcome Measures?

**Appendix B**

*Experimental spelling task*

**List A**

Regagner (to win back), incrédule (incredulous), surhomme (superman), parapente (paraglider), exclamer (to exclaim), arrestation (arrest), parfumeur (to perfume), honteux (ashamed), cordonnier (shoemaker), métallique (metallic), impertinent, irrespirable (unbreathable), déranger (to disturb), mésentente (discord), emprisonner (to imprison), souriceau (baby mouse), verdâtre (greenish), bénéfice (profit), planchette (board), semblable (similar)

**List B1**

Retirer (to pull back), infortune (misfortune), surplomb (overhang), parachute, extraire (to extract), consommation (consumption), rédacteur (writer), douteux (doubtful), conférencier (speaker), historique (historical), impensable (unthinkable), irréversible (irreversible), décoller (to unstick), mécontent (discontented), empaqueter (to wrap), pintadeau (young guinea fowl), brunâtre (brownish), artifice, clochette (small bell), classable (classifiable)

**List B2**

Remonter (to go back up), invalide, sursaut (jolt), paravent (screen), exposer (to exhibit), affectation (allocation), serviteur (servant), anxieux (anxious), couturier (dress designer), fantastique (fantastic), impersonnel (impersonal), irrésistible (irresistible), dévisser (unscrew), mésaventure (misadventure), empoisonner (to poison), baleineau (whale calf), grisâtre (grayish), maléfice (evil spell), plaquette (pack), gonflable (inflatable)
