# Supplementary material for: Derivational Morphology Training in French-Speaking 9- to 14- Year-Old Children and Adolescents With Developmental Dyslexia: Does It Improve Morphological Awareness, Reading, and Spelling Outcome Measures?
Source: J Learn Disabil. 2024 Feb 7;58(1):62–77. doi: 10.1177/00222194231223526 (PMC11636023; doi:10.1177/00222194231223526)
Supplement: sj-docx-3-ldx-10.1177_00222194231223526 – Supplemental material for Derivational Morphology Training in French-Speaking, 9- to 14- Year-Old Children and Adolescents With Developmental Dyslexia: Does it Improve Morphological Awaraness, Reading and Spelling Outcome Measures? [file sj-docx-3-ldx-10.1177_00222194231223526.docx]

**JOURNAL OF LEARNING DISABILITIES SUPPLEMENTAL FILE**

Derivational Morphology Training in French-Speaking, 9- to 14-Year-Old Children and Adolescents with Developmental Dyslexia: Does it Improve Morphological Awareness, Reading and Spelling Outcome Measures?

**Appendix C**

*Mean Number and Standard Deviation of Correct Whole Words (Base and Affix) in Spelling Tests for Each Group per Assessment Time. (T1, T2 and T3)*

|  | Measure | ITG^[[1]](#endnote-1)^ | | DTG^[[2]](#endnote-2)^ | |
| --- | --- | --- | --- | --- | --- |
|  |  | *M* | *SD* | *M* | *SD* |
| List A  Max = 20 | T1 (*n* = 82) | 4.37 | 3.08 | 5.07 | 4.47 |
|  | T2 (*n* = 82) | 10.29 | 4.51 | 5.17 | 3.51 |
|  | T3 (*n* = 76) | 9.30 | 3.84 | 5.78 | 3.71 |
| List B  Max = 20 | T1 (*n* = 82) | 4.71 | 2.87 | 5.66 | 4.27 |
|  | T2 (*n* = 82) | 9.20 | 3.71 | 6.40 | 3.46 |
|  | T3 (*n* = 76) | 9.11 | 3.42 | 6.94 | 4.01 |

1. ITG : Immediate training group [↑](#endnote-ref-1)
2. DG : Delayed training group [↑](#endnote-ref-2)
